# NMR data for novel flavonoids from *Lonicera japonica* flower buds

**DOI:** 10.1016/j.dib.2018.11.021

**Published:** 2018-11-10

**Authors:** Lanlan Ge, Jiemei Li, Haoqiang Wan, Keda Zhang, Weigang Wu, Xiaoting Zou, Shiping Wu, Boping Zhou, Jun Tian, Xiaobin Zeng

**Affiliations:** a Center Lab of Longhua Branch, Shenzhen People׳s Hospital, 2nd Clinical Medical College of Jinan University, Shenzhen 518120, Guangdong Province, China; b Department of Infectious Disease, Shenzhen People׳s Hospital, 2nd Clinical Medical College of Jinan University, Shenzhen 518120, Guangdong Province, China; c Integrated Chinese and Western Medicine Postdoctoral Research Station, Jinan University, Guangzhou 510632, Guangdong Province, China; d College of Life Science, Jiangsu Normal University, Xuzhou 221116, Jiangsu Province, China

## Abstract

The data presented in this article are associated with the research article entitled *“***Novel flavonoids from*****Lonicera japonica*****flower buds and validation of their anti**-**hepatoma and hepatoprotective activity*****in vitro*****studies***”* (Ge et al., 2018) [1]. The aim of this data was to provide the NMR spectrum of novel flavonoids from *Lonicera japonica* flower buds. Samples were isolated from EtOAc fraction of *Lonicera japonica* flower buds extracts, then dissolved in DMSO-d_6_ before NMR testing.

## Specifications table

**Table:** 

Subject area	*Chemistry*
More specific subject area	*Natural products research*
Type of data	*Figure, Table*
How data was acquired	*NMR of flavonoids*
Data format	*Filtered and Analyzed*
Experimental factors	*First, the samples were isolated from EtOAc fraction of Lonicera japonica flower buds extracts. Then the samples were dissolved in d*_*6*_*-DMSO before NMR testing.*
Experimental features	*Nuclear magnetic resonance (NMR) spectra data of flavonoids from L. japonica flower buds were recorded on a Bruker DPX-400 spectrometer using standard Bruker pulse programs (Bruker, Karlsruhe, Germany). Chemical shifts were shown as δ-values with reference to tetramethylsilane (TMS) as an internal standard.*
Data source location	*Center Lab of Longhua Branch, Shenzhen People׳s Hospital, 2nd Clinical Medical College of Jinan University, Shenzhen, Guangdong Province, China.*
Data accessibility	*Data is with this article*
Related research article	Ge LL, Li JM, Wan HQ, Zhang KD, Wu WG, Zou XT, Wu SP, Zhou BP, Tian J, Zeng XB. Novel flavonoids from *Lonicera japonica* flower buds and validation of their anti-hepatoma and hepatoprotective activity *in vitro* studies. Industrial Crops and Products. 2018 125: 114–122 [1].

## Value of the data

• NMR data of flavonoids is useful for elucidating their chemical structures.
• Method and data provide useful information for isolating novel flavonoids.

## 1 Data

Fourteen flavonoids were isolated and identified from *Lonicera japonica* flower buds. Besides, compounds **2**–**3**, **5**, **12** were new compounds, renamed japoflavone A–D. The NMR data of japoflavone A and B suggest they were structure analogue, which were composed of a flavonoid unit and several aromatic ring groups. Japoflavone C was 5-hydroxy-7,3׳,5׳-trimethoxyflavone. Japoflavone D was a biflavonoid structure with a C–C bond. Four novel flavonoids were first isolated and identified from *L. japonica* flower buds. See Fig. 1, Fig. 2, Fig. 3, Fig. 4, Fig. 5, Fig. 6, Fig. 7, Fig. 8, Fig. 9, Fig. 10, Fig. 11, Fig. 12, Fig. 13, Fig. 14, Fig. 15, Fig. 16, Fig. 17, Fig. 18, Fig. 19, Fig. 20, Fig. 21, Fig. 22, Fig. 23, Fig. 24, Fig. 25.

**Fig. 1:**
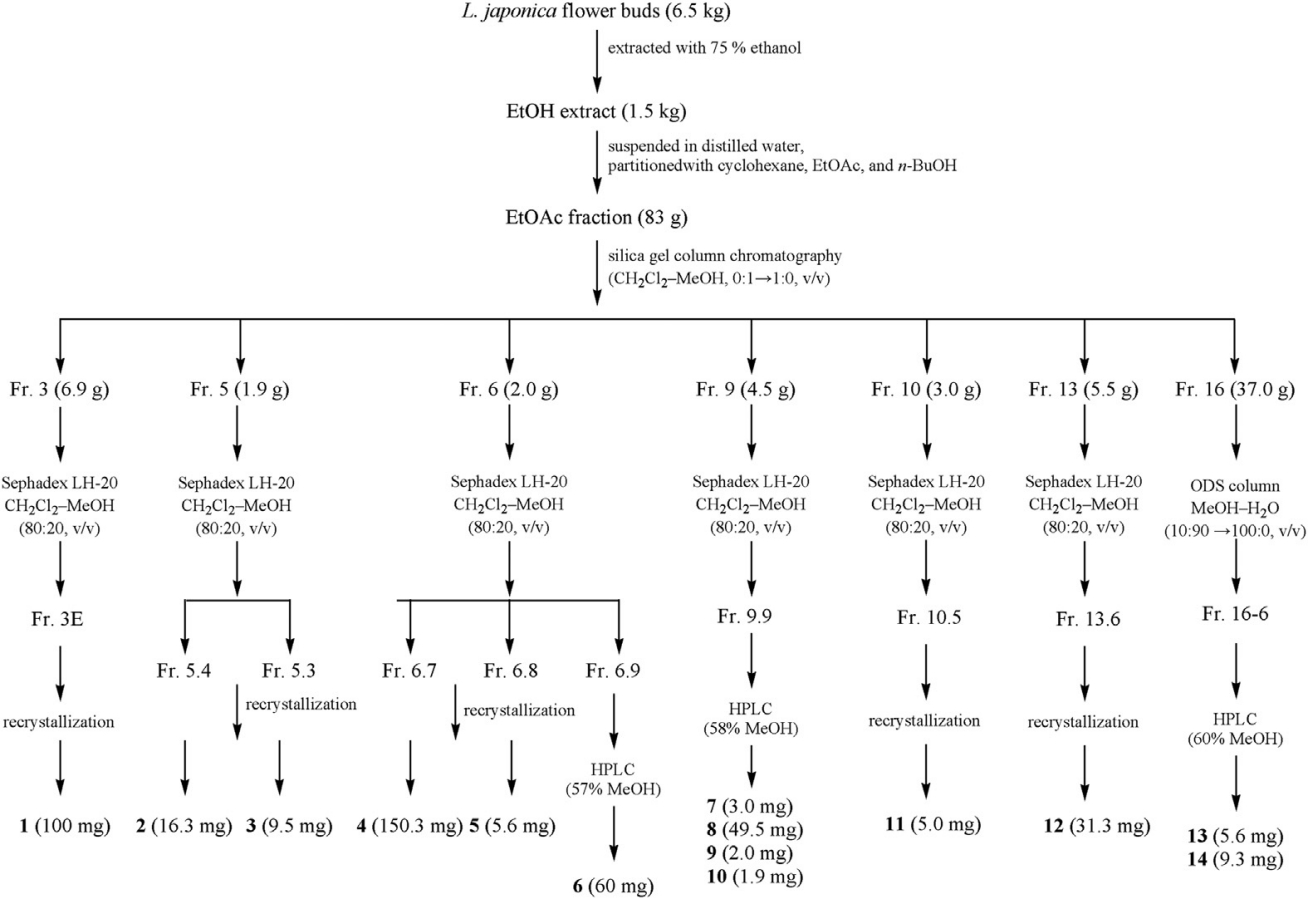
Extraction and isolation procedure of flavonoids from *L. japonica* flower buds.

**Fig. 2:**
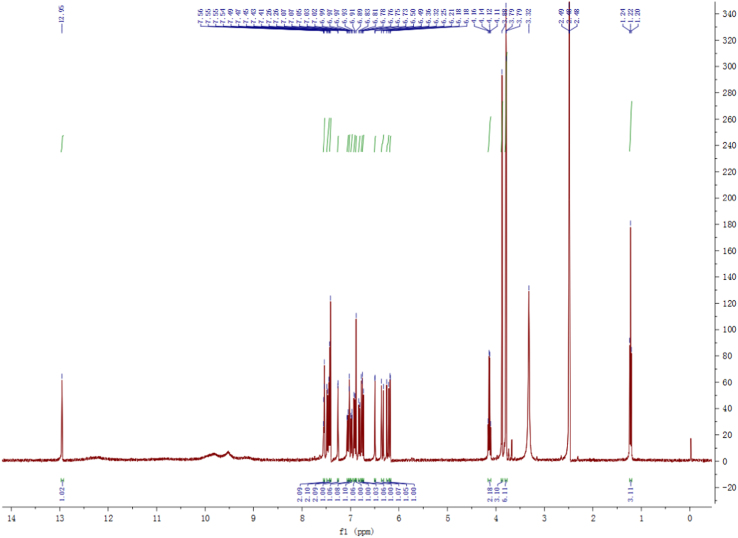
^1^H-NMR spectrum of compound **2**.

**Fig. 3:**
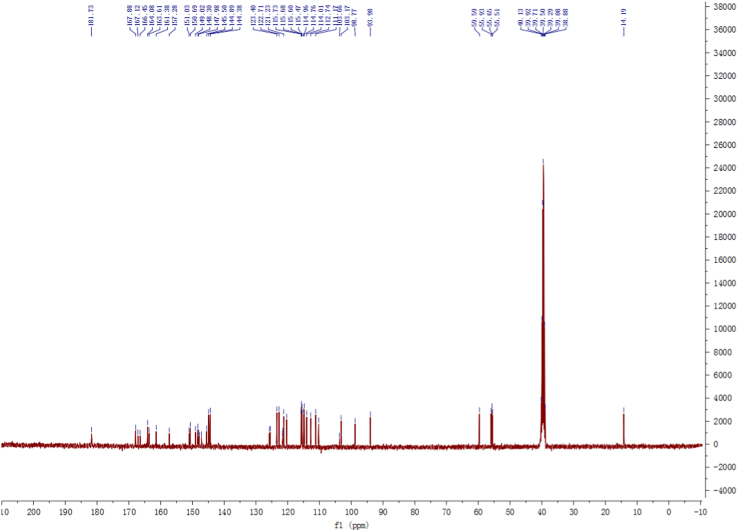
^13^C-NMR and DEPT spectrum of compound **2**.

**Fig. 4:**
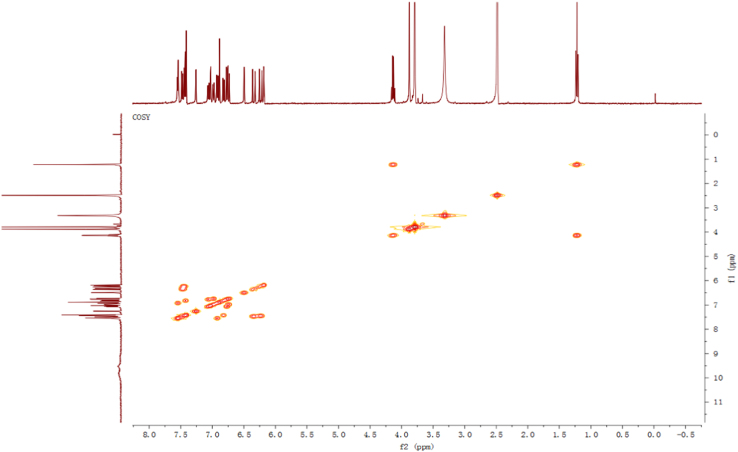
^1^H-^1^H COSY spectrum of compound **2**.

**Fig. 5:**
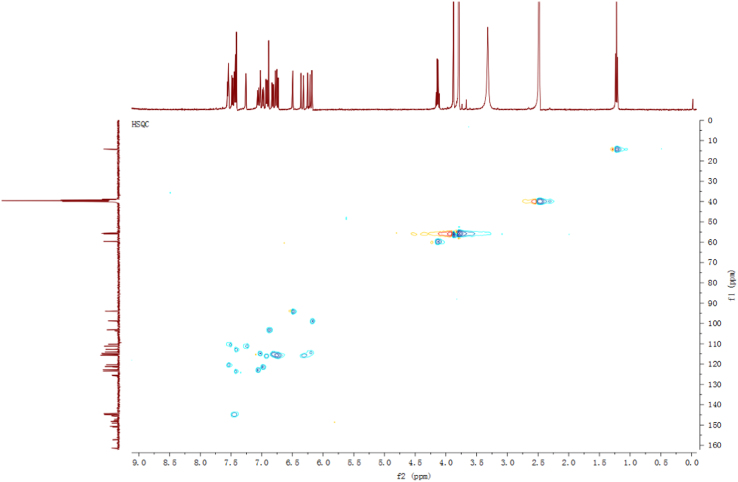
HSQC spectrum of compound **2**.

**Fig. 6:**
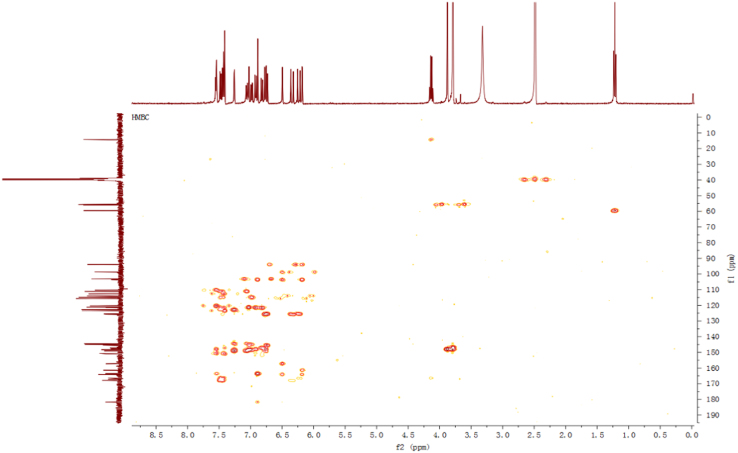
HMBC spectrum of compound **2**.

**Fig. 7:**
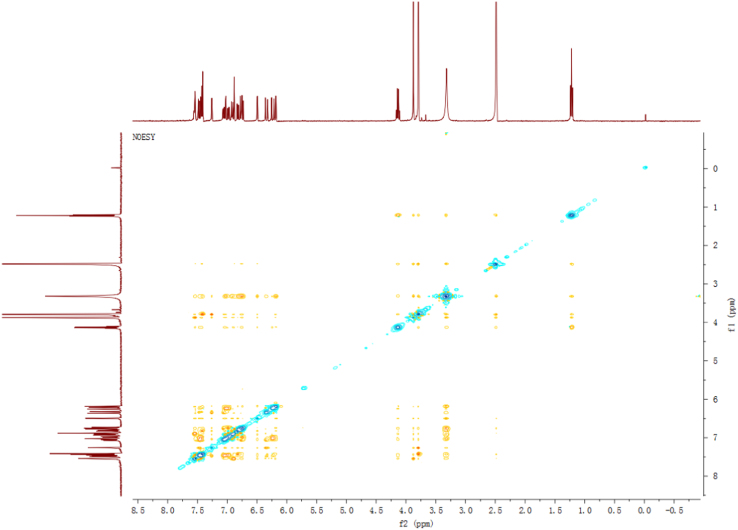
NOESY spectrum of compound **2**.

**Fig. 8:**
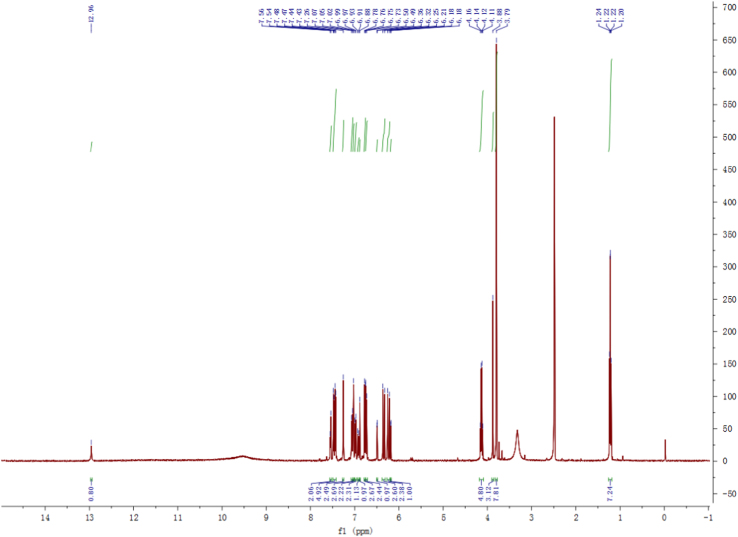
^1^H-NMR spectrum of compound **3**.

**Fig. 9:**
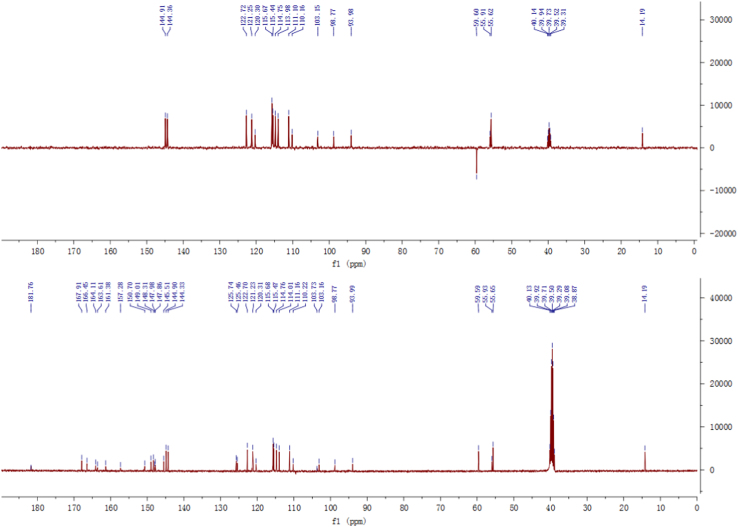
^13^C-NMR and DEPT spectrum of compound **3**.

**Fig. 10:**
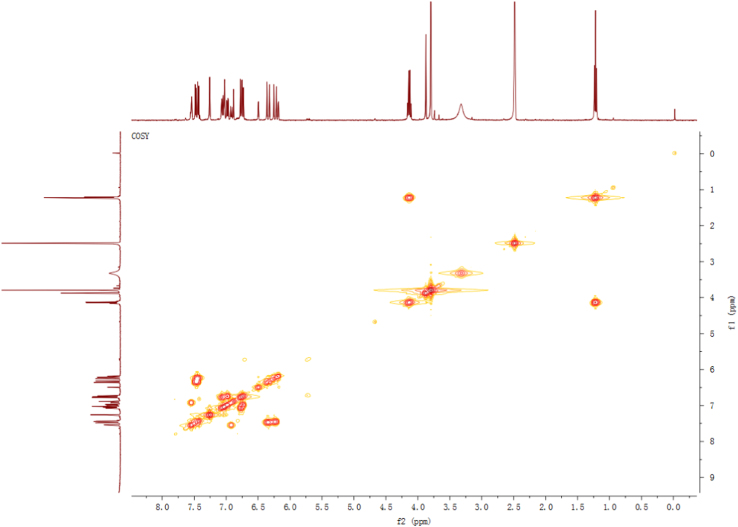
^1^H–^1^H COSY spectrum of compound **3**.

**Fig. 11:**
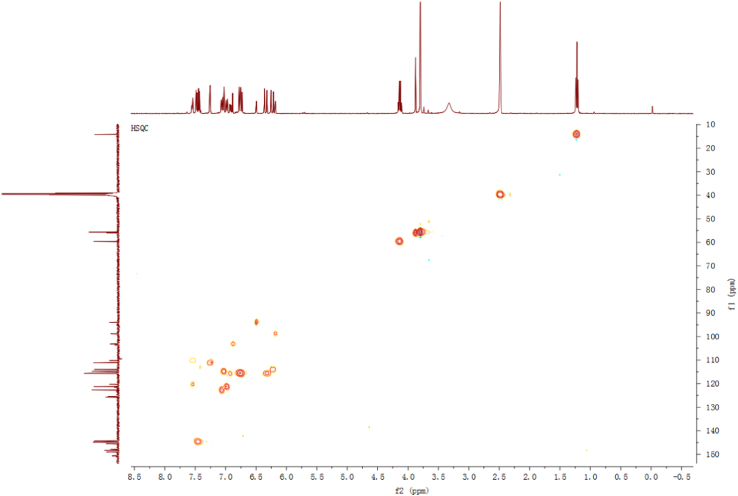
HSQC spectrum of compound **3**.

**Fig. 12:**
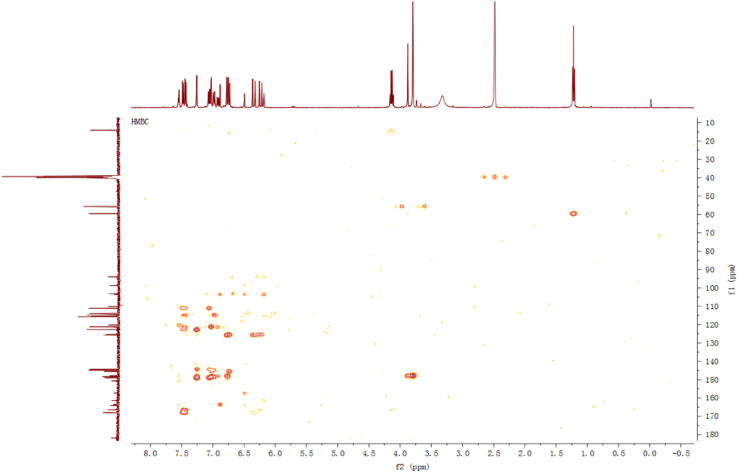
HMBC spectrum of compound **3**.

**Fig. 13:**
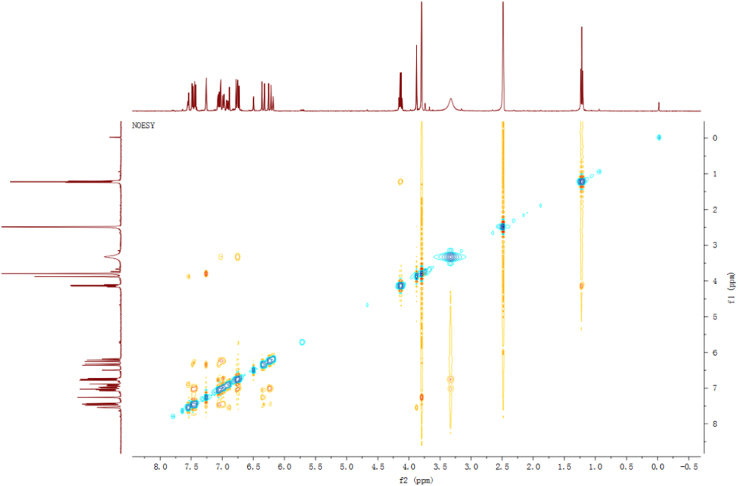
NOESY spectrum of compound **3**.

**Fig. 14:**
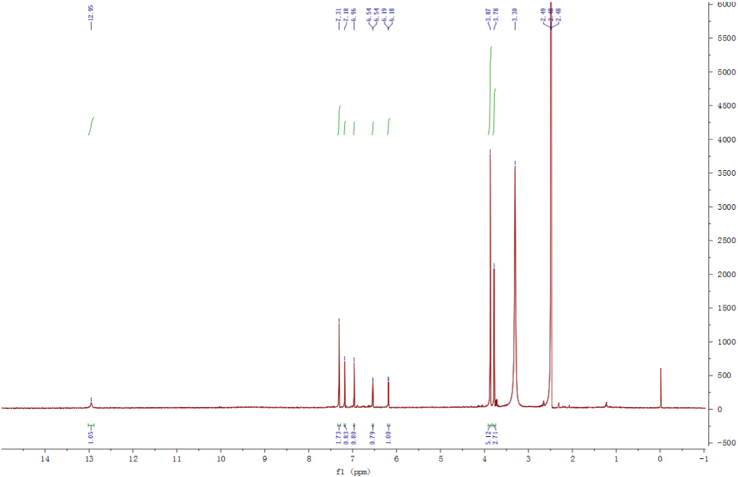
^1^H-NMR spectrum of compound **5**.

**Fig. 15:**
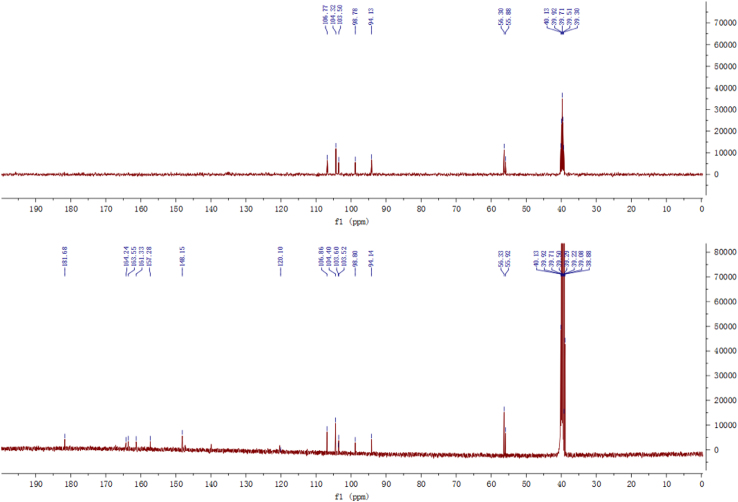
^13^C-NMR and DEPT spectrum of compound **5**.

**Fig. 16:**
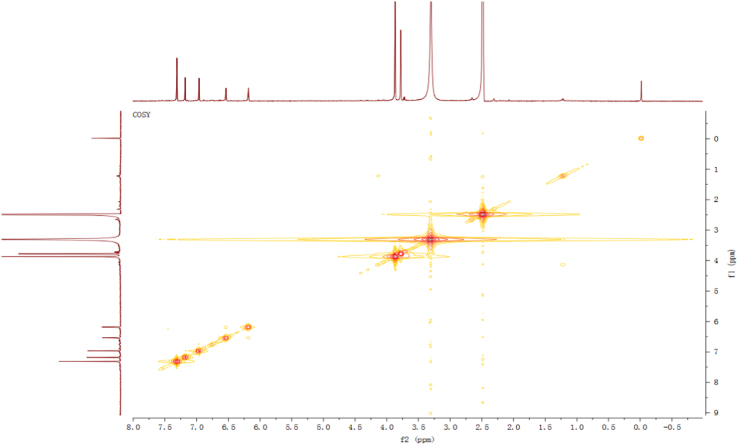
^1^H–^1^H COSY spectrum of compound **5**.

**Fig. 17:**
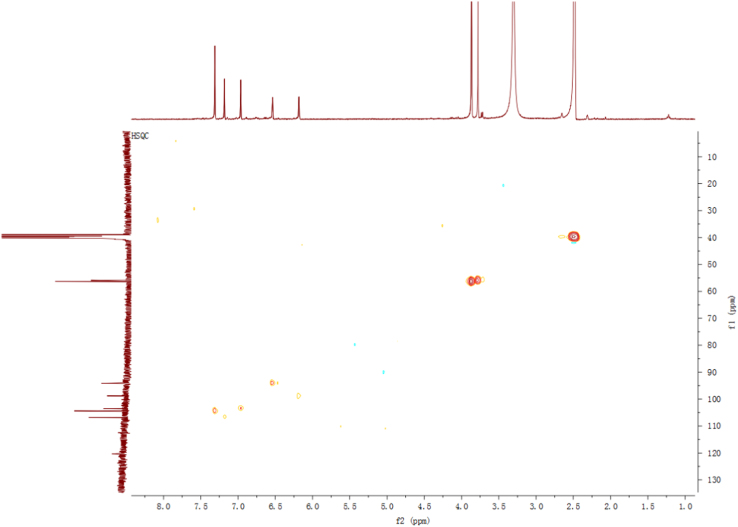
HSQC spectrum of compound **5**.

**Fig. 18:**
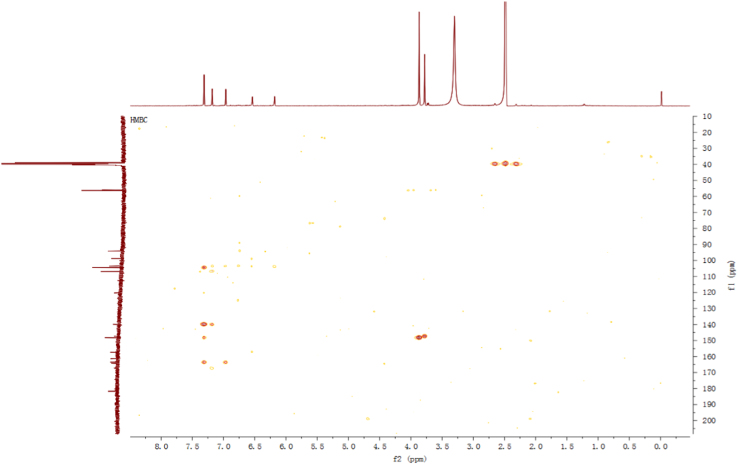
HMBC spectrum of compound **5**.

**Fig. 19:**
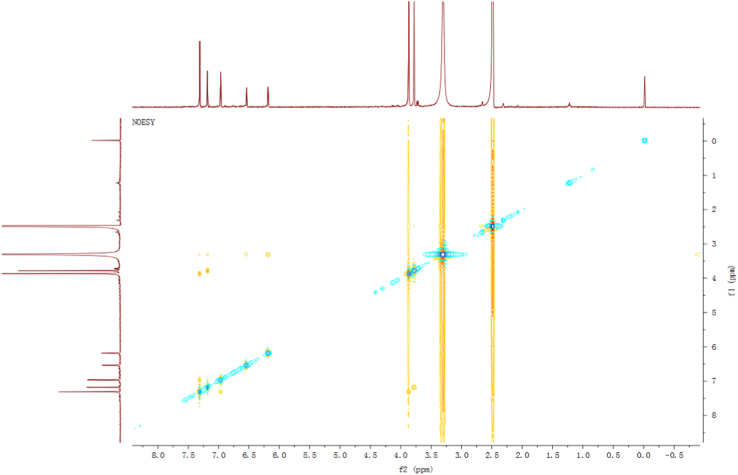
NOESY spectrum of compound **5**.

**Fig. 20:**
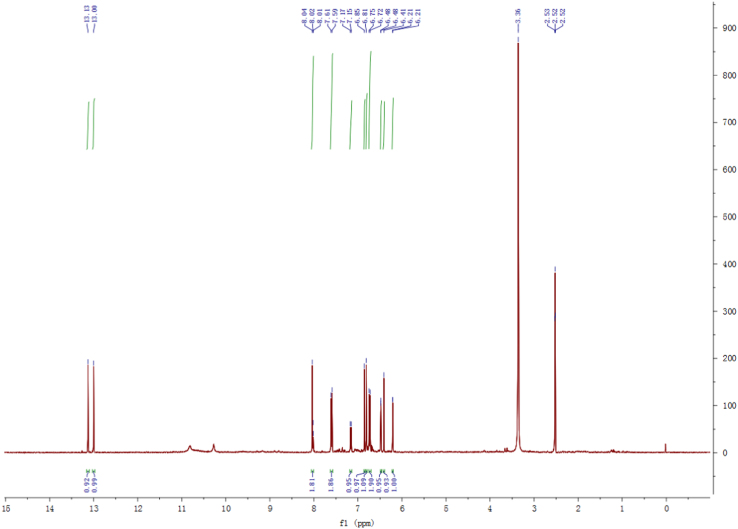
^1^H-NMR spectrum of compound **12**.

**Fig. 21:**
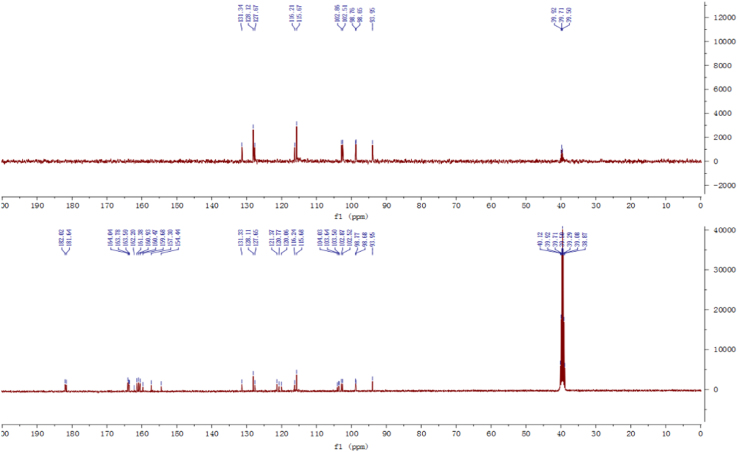
^13^C-NMR spectrum of compound **12**.

**Fig. 22:**
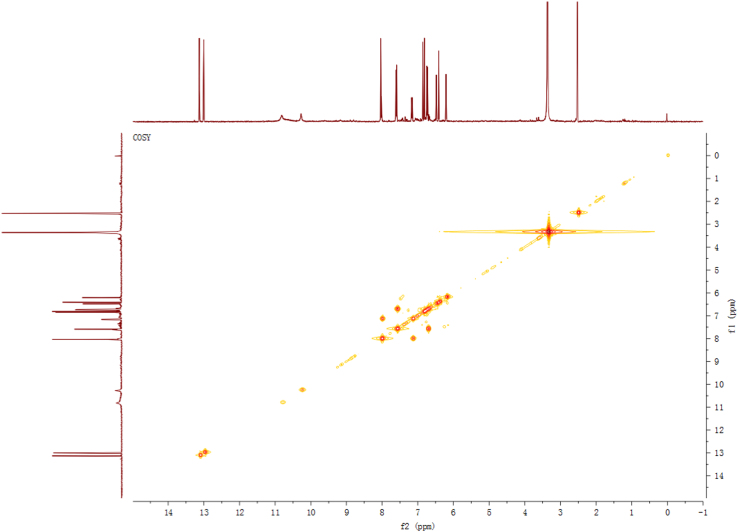
^1^H–^1^H COSY spectrum of compound **12**.

**Fig. 23:**
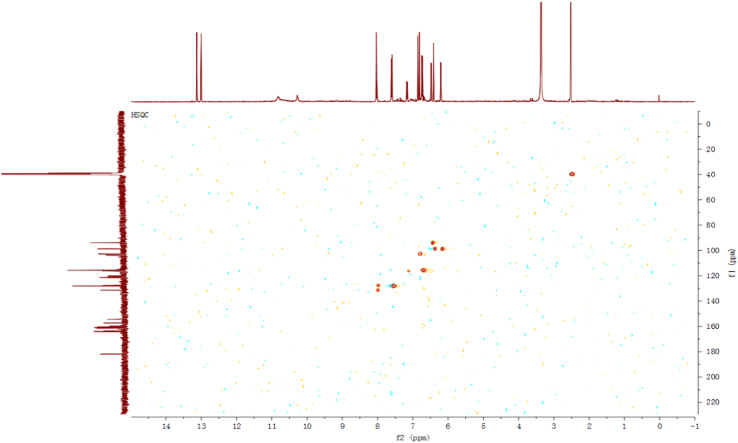
HSQC spectrum of compound **12**.

**Fig. 24:**
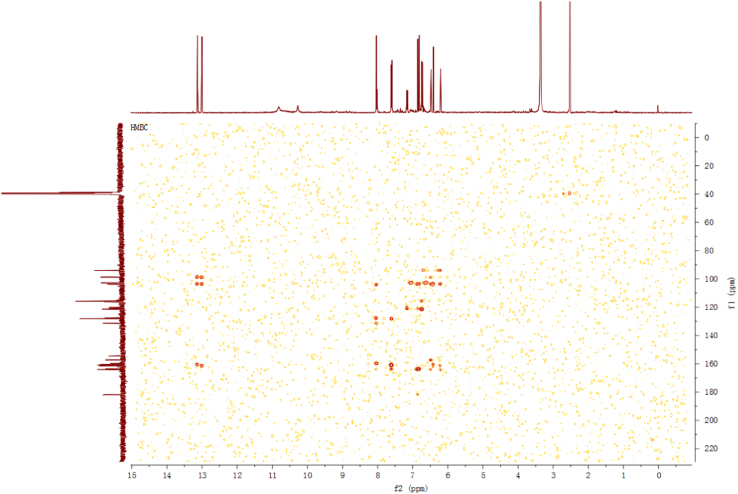
HMBC spectrum of compound **12**.

**Fig. 25:**
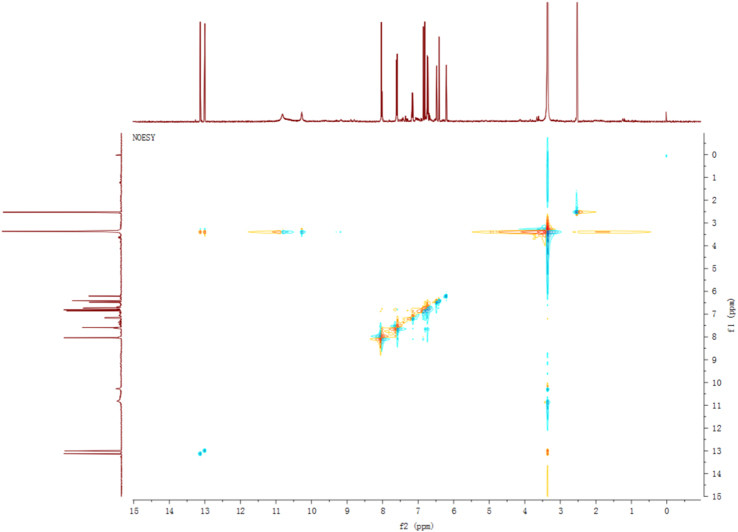
NOESY spectrum of compound **12**.

## 2 Experimental design, materials, and methods

### 2.1 Study area description

Jin Yin Hua, the flower buds of *Lonicera japonica* Thunb. (Caprifoliaceae), have been used for treating influenza, cold, fever, and infections [2], [3]. On this basis, we chose *L. japonica* flower buds as the experimental material [1]. To systematically study the flavonoids in *L. japonica* flower buds, fourteen flavonoids were isolated by solvent extraction, silica gel, Sephadex LH-20, ODS, and preparative high-performance liquid chromatography (HPLC). Their structures were identified by various chromatography methods including NMR and MS spectrum.

### 2.2 Sample collection

The *L. japonica* flower buds were collected in May 2017 from Pingyi County, Linyi City, Shandong Province, China (N 35°30׳22.33׳׳ E 117°38׳5.13׳׳).

### 2.3 Extraction and isolation

The extraction and isolation procedure of fourteen flavonoids from *L. japonica* flower buds is shown in Fig. 1.

### 2.4 NMR spectrum of japoflavone A–D

In addition to japoflavone A–D, ten known compounds were isolated from *L. japonica* flowers. The known compounds were identified as 5-hydroxy-7,3׳,4׳-trimethoxyflavone (**1**) [4], tricin (**4**) [5], chrysoeriol (**6**) [6], eriodictyol (**7**) [7], luteolin (**8**) [8], kaempferol (**9**) [9], apigenin (**10**) [10], quercetin (**11**) [11], astragalin (**13**) [11], and quercetin-3-O-*β*-D-6 (*p*-coumaroyl) glucopyranoside (**14**) [12].

#### 2.4.1 Japoflavone A (2)

Pale yellow, amorphous power; 1D and 2D NMR spectrum see Fig. 2, Fig. 3, Fig. 4, Fig. 5, Fig. 6, Fig. 7.

#### 2.4.2 Japoflavone B (3)

Yellow, amorphous power; 1D and 2D NMR spectrum see Fig. 8, Fig. 9, Fig. 10, Fig. 11, Fig. 12, Fig. 13.

#### 2.4.3 Japoflavone C (5)

Yellow amorphous power; 1D and 2D NMR spectrum see Fig. 14, Fig. 15, Fig. 16, Fig. 17, Fig. 18, Fig. 19.

#### 2.4.4 Japoflavone D (12)

Yellow amorphous power; 1D and 2D NMR spectrum see Fig. 20, Fig. 21, Fig. 22, Fig. 23, Fig. 24, Fig. 25.
