# Supplementary material for: NMR data for novel flavonoids from Lonicera japonica flower buds
Source: Data Brief. 2018 Nov 10;21:2192–207. doi: 10.1016/j.dib.2018.11.021 (PMC6276635; doi:10.1016/j.dib.2018.11.021)
Supplement: Supplementary file 1 — Supplementary material [file mmc1.doc]

**Conflict of Interest Form**

The authors have declared that there is no conflict of interest.
